# Supplementary material for: Snapshot of Phenotypic and Molecular Virulence and Resistance Profiles in Multidrug-Resistant Strains Isolated in a Tertiary Hospital in Romania
Source: Pathogens. 2023 Apr 17;12(4):609. doi: 10.3390/pathogens12040609 (PMC10145626; doi:10.3390/pathogens12040609)
Supplement: Supplementary file 1 [file pathogens-12-00609-s001.zip › pathogens-2344205-supplementary.pdf]

pathogens

MDPI

Supplementary materials

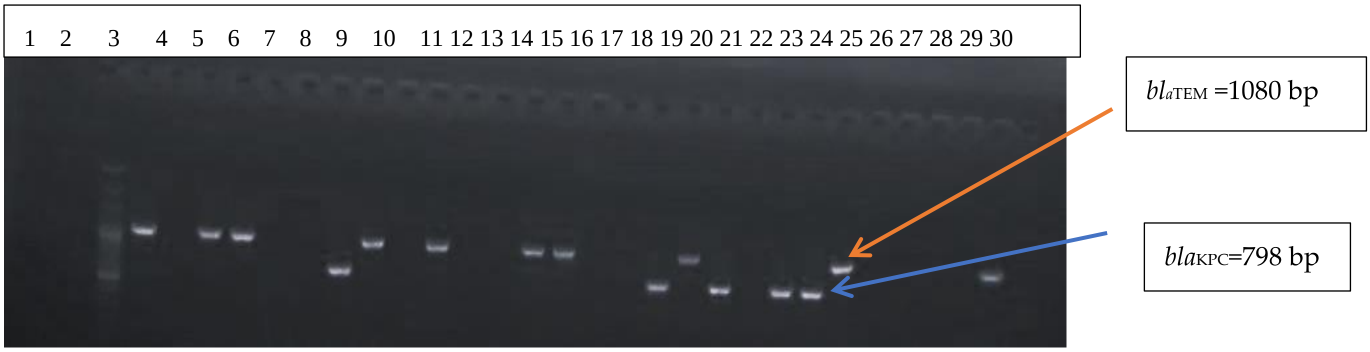

Figure S1. Electrophoresis gel for *bla*TEM (1080 bp) and *bla*KPC (798 bp) genes detection in *K. pneumoniae* strains. Lines: **1**-Molecular Size Marker (ThermoScientific, 1500 bp); **2-** strain 1; **3-** 3; **4-** 8; **5-** 11; **6-** 12; **7-** 13; **8-** 14; **9-** 15; **10-** 17; **11-** 19; **12-** 21; **13-** 24; **14-** 25; **15-** 26; **16-** 27; **17-** 28; **18-** 29; **19-** 30; **20-** 34; **21-** 37; **22-** 38; **23-** *K. pneumoniae* positive control (for *bla*KPC); **24-** 47; **25-** 48; **26-** 49; **27-** 50; **28-** 51; **29-** *K. pneumoniae* positive control (for *bla*TEM); **30-** 106. Positive strains for *bla*TEM gene- 1; 8; 11 ; 15 ; 19 25 ; 26 ; 30; 47; positive control. Positive strains for *bla*KPC gene- 14; 29; 34 ; 38 ; positive control.

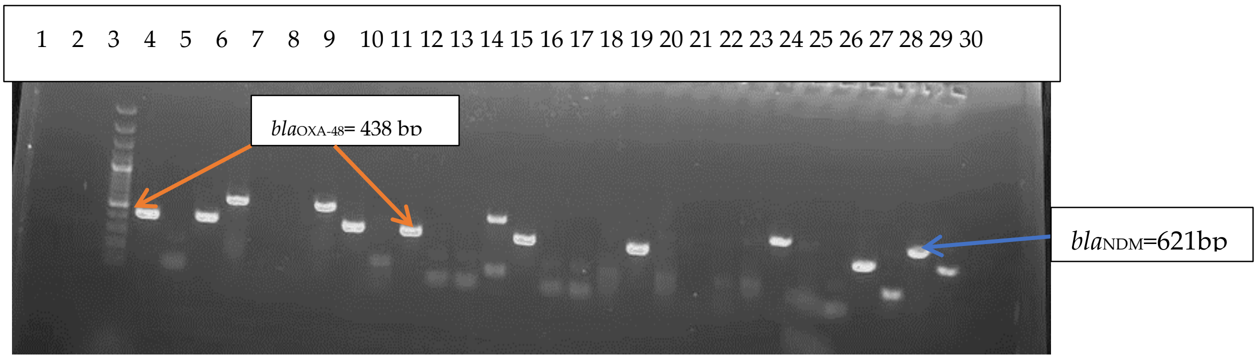

Figure S2. Electrophoresis gel for *bla*OXA-48 (438 bp) and *bla*NDM (621 bp) genes detection in *K. pneumoniae* strains. Lines: **1**- Molecular Size Marker (ThermoScientific, 1500 bp); **2-** strain 1; **3-** 3; **4-** 8; **5-** 11 ; **6-** 12; **7-** 13; **8-** 14; **9-** 15; **10-** 17; **11-** 19; **12-** 21; **13-** 24; **14-** 25; **15-** 26; **16-** 27; **17-** 28; **18-** 29; **19-** 30; **20-** 34; **21-** 37; **22-** 38; **23-** 43; **24-** 47; **25-** 48; **26-** positive control (for *bla*OXA-48 gene); **27-** 50; **28-** positive control (for *bla*NDM gene); 29-52; **30-** 106. Positive strains for *bla*NDM gene- 11; 14; 25; 43; positive control. Positive strains for *bla*OXA-48 gene- 1; 8; 15; 17; positive control; 29; 52.

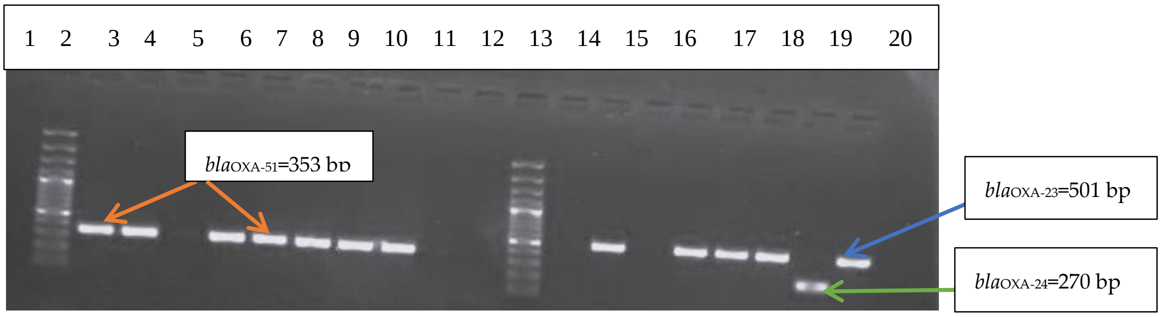

Figure S3. Electrophoresis gel for *bla*OXA-51 (346 bp), *bla*OXA-23(501 bp) and *bla*OXA-24 (242 bp) genes detection in *A. baumannii* strains. Lines: **1-** Molecular Size Marker (ThermoScientific, 1500 bp); **2-** strain *A. baumannii* positive control for

bla_{OXA-51} gene ; **3-** 3A51; **4-** 17A51; **5-** 21 A51; **6-** 24 A51; **7-** 27 A51; **8**- 28 A51; **9**- 49A; **10**- 35S (negative control); **11-** 51; **12**- Molecular Size Marker (ThermoScientific, 1500 bp) ; **13**- Acc; **14**- *A. baumannii* positive control (for bla_{OXA-23}); **15**- 17A3; **16**- 21A3; **17**- 24A3; **18**- 27 A3; **19**- *A. baumannii* positive control (for bla_{OXA-24}); **20**- 49 A3. Positive strains for bla_{OXA-51} - positive control; 3A51; 21 A51; 24 A51; 27 A51; 28 A51; 49 A. Positive strains for bla_{OXA-23}- positive control; 21A3; 24A3; 27 A3; 49A3. Positive strains for bla_{OXA-24}- positive control.

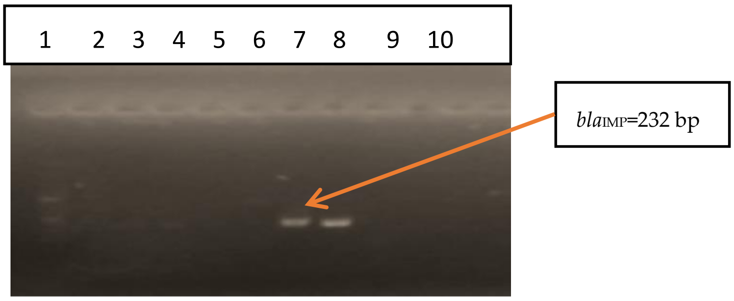

Figure S4. Electrophoresis gel for bla_{IMP} gene (232 bp) detection in *P. aeruginosa* strains. Lines: **1**-Molecular Size Marker (ThermoScientific, 1500 bp); 2- strain 12; **3-** 37; **4-** 81; **5-** 82; **6-** 88; **7-** 92**;** **8**- *P. aeruginosa* positive control for bla_{IMP} gene; 9-98; **10**- negativ control for bla_{IMP} gene. Positive strains for bla_{IMP} gene- 92; positive control.

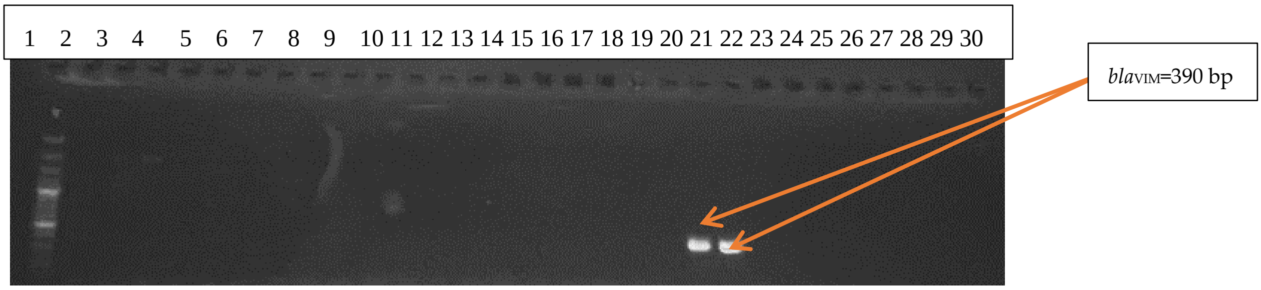

Figure S5. Electrophoresis gel for $bla_{VIMlike}$ (390 bp) gene detection: one strain of *P. aeruginosa* encoded 81 was positive and the positive control. Lines: **1**- Molecular Size Marker (ThermoScientific, 1500 bp); **2-** strain 105; **3-** 107; **4-** 110; **5-** 97; **6-** 98; **7-** 100; **8**- 64; **9**- 66; **10**- 72; **11-** 112; **12**- 54; **13**- 59; **14**- 60; **15**- 61; **16**- 67; **17**- 35; **18**- Acc; **19**- 78; **20**- 79; **21**- 81; **22**- *P. aeruginosa* positive control for $bla_{VIMlike}$ gene; **23**- 88; **24**- 92; **25**- 94; **26**- 95; **27**- 86; **28**- PSB; **29**- SA; **30**- negativ control.

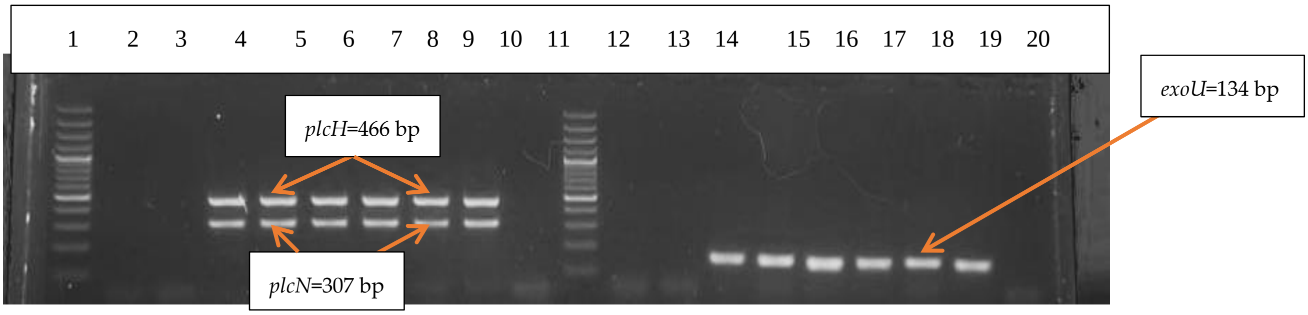

Figure S6. Electrophoresis gel for *plcN* (307 bp), *plcH* (466 bp), *exoU* (134 bp) and *exoT* (152 bp) genes detection in *P. aeruginosa* strains. Lines: **1**- Molecular Size Marker (ThermoScientific, 1500 bp); **2 –** strain 12; **3-** 37; **4-** 81; **5-** 82; **6-** 88; **7-** 92; **8**- 98; **9**- *P. aeruginosa* positive control for *plcH* and *plcN* genes; **10**- negativ control for *plcH* and *plcN* genes; **11-** Molecular Size Marker (ThermoScientific, 1500 bp); **12**- 12; **13**- 37; **14**- 81; **15**- 82; **16**- 88; **17**- 92; **18**- 98; **19**- *P. aeruginosa* positive control for *exoU* gene; **20**- negativ control for *exoU* gene. Positive strains for *plcH/N* genes- 81; 82; 88; 92;98; positive control. Positive strains for *exoU* gene- 81; 82; 88; 92; 98; positive control.

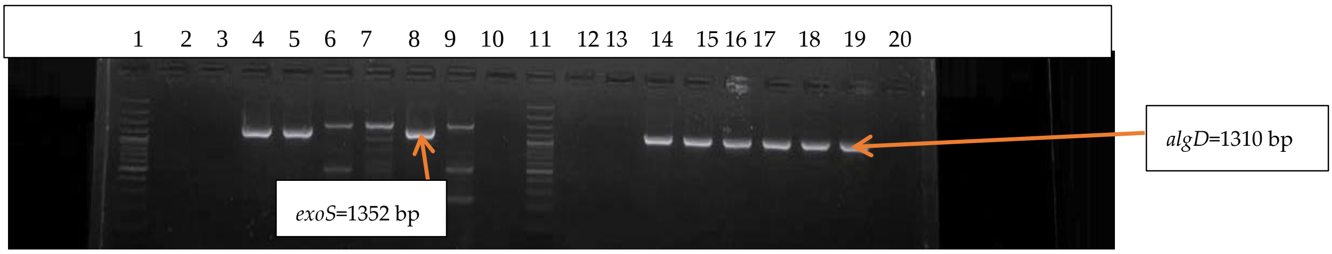

Figure S7. Electrophoresis gel for *exoS* (1352 bp) and *algD* (1310 bp) genes detection in *P. aeruginosa* strains. Lines: **1**- Molecular Size Marker (ThermoScientific, 1500 bp); **2-** strain 12; **3-** 37; **4- 81**; **5-** 82; **6-** 88; **7-** 92; **8-** *P. aeruginosa* positive control for *exoS* gene; **9**- PsB; **10**- negativ control for *exoS* gene; **11-** Molecular Size Marker (ThermoScientific, 1500 bp); **12**- 12; **13**- 37; **14**- 81; **15**- 82; **16**- 88; **17**- 92; **18**- 98; **19**- *P. aeruginosa* positive control for *algD* gene; **20**- negativ control for *algD* gene. Positive strains for *exoS* gene- 81; 82; positive control. Positive strains for *algD* gene- 81; 82; 88; 92; 98; positive control.

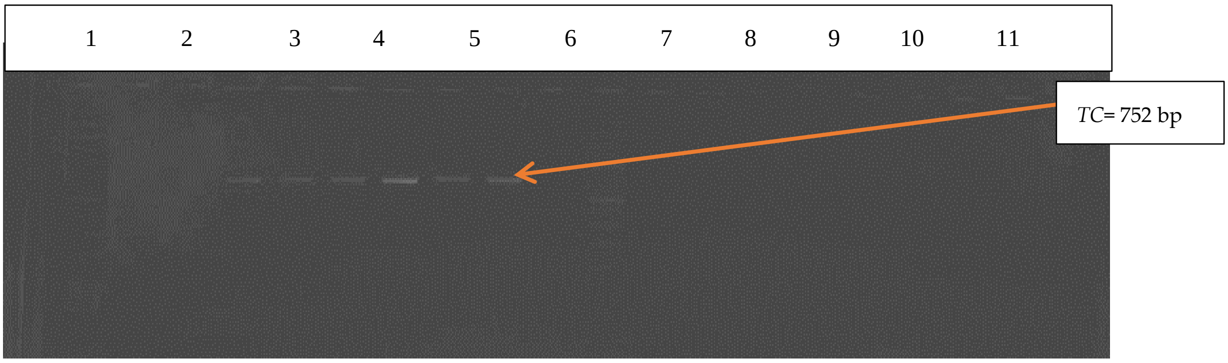

Figure S8. Electrophoresis gel for *TC* gene (752 bp) detection in *P. aeruginosa* strains. Lines: **1**- Molecular Size Marker (ThermoScientific, 1500 bp); **3-** strain 37; **4- 81**; **5-** 82; **6-** 88; **7-** 92; **8**- 98; **9**- *P. aeruginosa* positive control for *TC* gene; **10**- negativ control for *TC* gene; **11-** Molecular Size Marker (ThermoScientific, 1500 bp). Positive strains for *TC* gene- 81; 82; 88; 92; 98; positive control.

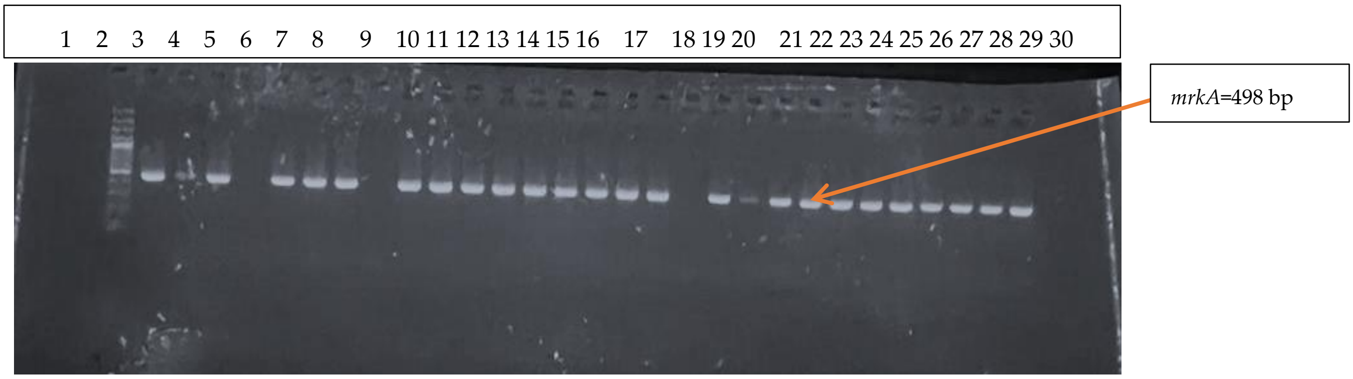

Figure S9. Electrophoresis gel for *mrkA* (498 bp) and *mrkD* (756 bp) genes detection in *K. pneumoniae* strains. Lines: **1**- Molecular Size Marker (ThermoScientific, 1500 bp); **2**-strain 1; **3-** 8; **4-** 11 ; **5-** 13; **6-** 14; **7-** 15; **8**- 19; **9**- 25; **10**- 26; **11-** 29; **12**- 34; **13**- 38; **14**- 43; **15**- 47; **16**- 49; **17**- 50; **18**- 51; **19**- 52; **20**- 54; **21**- 59; **22**- 60; **23**- 61; **24**- 64; **25**- 66; **26**- 72; **27**- 76; **28**- 78; **29**- 79; **30**- *K. pneumoniae* positive control for *mrkA* gene. Positive strains for *mrkA* gene- 1; 8; 11;14; 15; 19; 26; 29; 34; 38; 43; 47; 49; 50; 51; 54; 59; 50; 61; 64; 66; 72; 76; 78; 79; positive control. All strains are negative for *mrkD* gene.

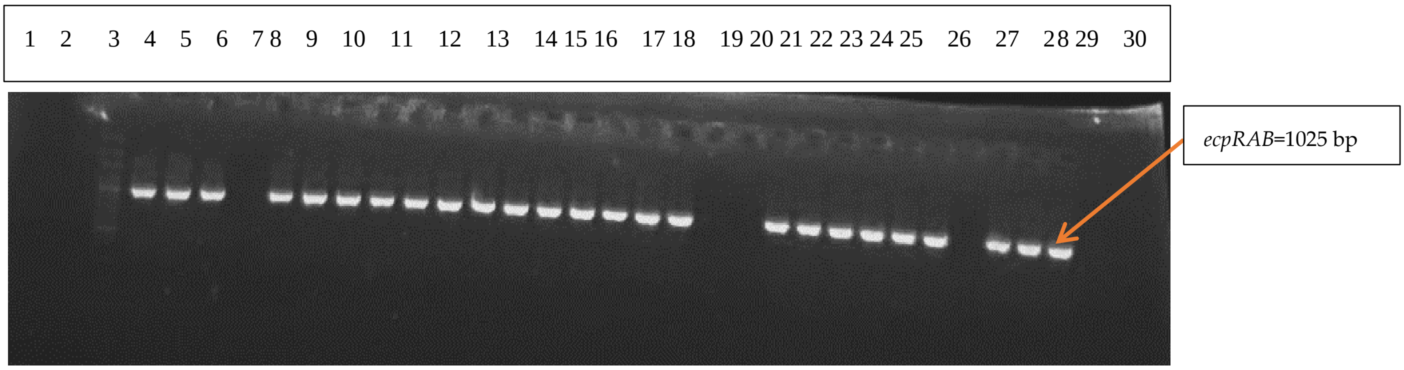

Figure S10. Electrophoresis gel for *ecpRAB* (1025 bp) gene detection in *K. pneumoniae* strains. Lines: **1**- Molecular Size Marker (ThermoScientific, 1500 bp); **2-** strain 1; **3-** 8; **4-** 11; **5-** 13; **6-** 14; **7-** 15; **8**- 19; **9**- 25; **10**- 26; **11-** 29; **12**- 30; **13**- 34; **14**-38; **15**- 43; **16**- 47; **17**- 48; **18**- 50; **19**- 51; **20**- 52; **21**- 54; **22**- 59; **23**- 60; **24**- 61; **25**- 69; **26**- 66; **27**- 72; **28**- 76; **29**- 78; **30**- *K. pneumoniae* positive control for *ecpRAB* gene. Positive strains for *ecpRAB* gene- 1;8; 11; 13; 15; 19; 25; 26; 29; 30; 34; 38; 43; 47; 48; 50; 54; 59; 60; 61; 69; 66; 76; 78; positive control.

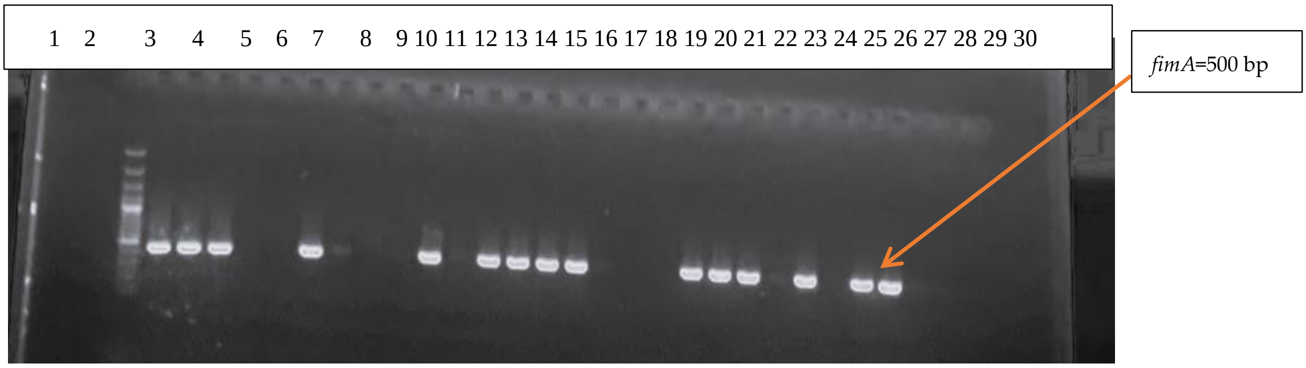

Figure S11. Electrophoresis gel for *fimA* (500 bp) gene detection in *K. pneumoniae* strains. Lines: **1**- Molecular Size Marker (ThermoScientific, 1500 bp); **2-** strain 1; **3-** 8; **4-** 11; **5-** 13; **6-** 14; **7-** 15; **8**- 19; **9**- 25; **10**- 26; **11-** 29; **12**- 30; **13**- 34; **14**-38; **15**- 43; **16**- 47; **17**- 48; **18**- 50; **19**- 51; **20**- 52; **21**- 54; **22**- 59; **23**- 60; **24**- 61; **25**- 64; **26**- 66; **27**- *K. pneumoniae* positive control for *fimA* gene; **28**- 76; **29**- 78; **30**- 79. Positive strains for *fimA* gene- 1; 8; 11; 15; 29; 34; 38; 43; 47; 52; 54; 59; 61; 66; positive control.

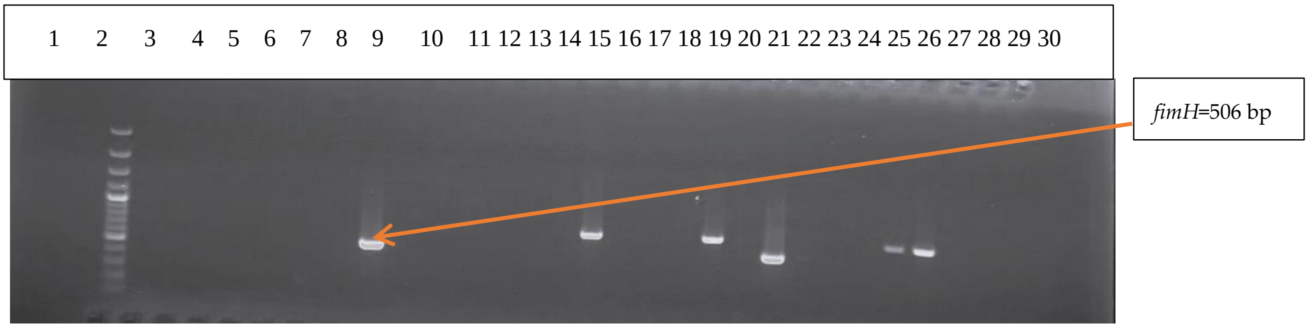

Figure S12. Electrophoresis gel for *fimH* (506 bp) gene detection in *K. pneumoniae* strains. Lines: **1**- Molecular Size Marker (ThermoScientific, 1500 bp); **2-** strain 1; **3-** 8; **4-** 11; **5-** 13; **6-** 14; **7-** 15; **8**-19; **9**- 25; **10**- 26; **11-** 29; **12**- 30; **13**- 34; **14**-

38; **15**- 43; **16**- 47; **17**- 48; **18**- 50; **19**- 51; **20**- 52; **21**- 54; **22**- *K. pneumoniae* positive control for *fimH* gene; **23**- 60; **24**- 61; **25**- 64; **26**-66; **27**- 72; **28**- 76; **29**- 78; **30**- 79. Positive strains for *fimH* gene- 25; positive control.

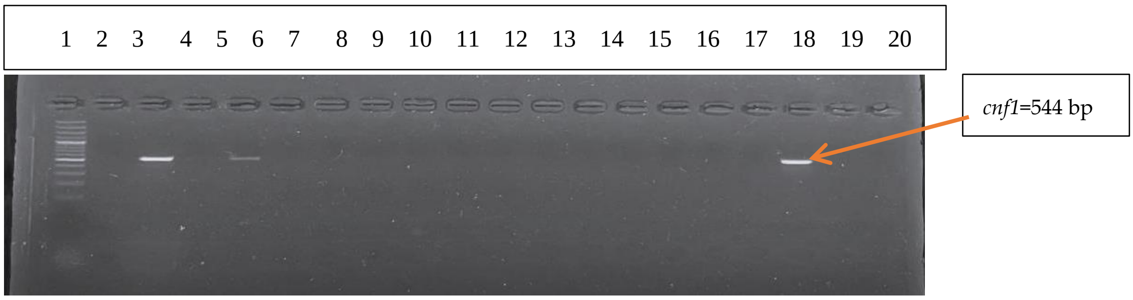

Figure S13. Electrophoresis gel for *cnf1* (544bp) gene detection in *K. pneumoniae* strains. Lines: **1**- Molecular Size Marker (ThermoScientific, 1500 bp); **2-** strain 54; **3-** 59; **4-** 60; **5-** 61; **6-** 64; **7-** 66; **8**- 72; **9**- 76; **10**- 78; **11-** 79; **12**- 86; **13**- 94; **14**- 95; **15**- 97; **16**- 100; **17**- 105; **18**- *K. pneumoniae* positive strain for *cnf1* gene; **19**-107; **20**- 110. Positive strains for *cnf1* gene - 59; positive control.

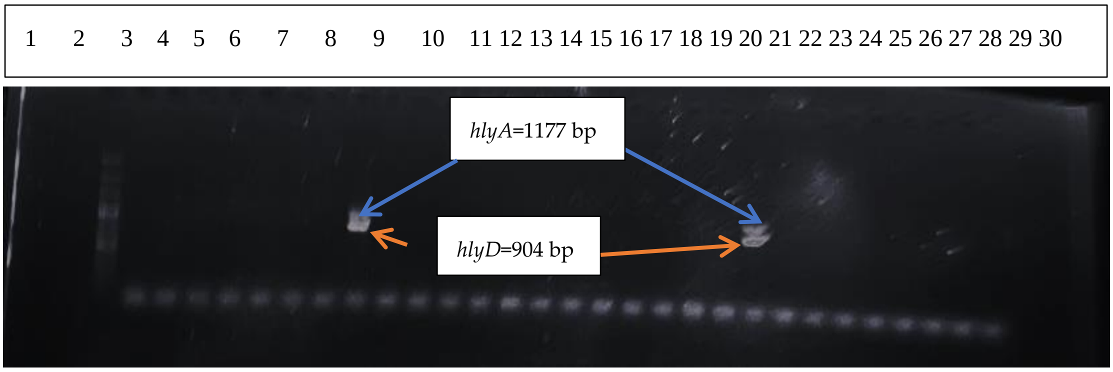

Figure S14. Electrophoresis gel for *hlyA* (1177 bp) and *hlyD* (904 bp) genes detection in *K. pneumoniae* strains. Lines: **1**- Molecular Size Marker (ThermoScientific, 1500 bp); **2-** strain 8; **3-** 11; **4-** 13; **5-** 14; **6-** 15; **7-** 19; **8**- 25; **9**- 26; **10**- 29; **11-** 30; **12**- 34; **13**- 38; **14**- 43; **15**- 47; **16**- 48; **17**- 50; **18**- 51; **19**- 51; **20**- 54; **21**- 59; **22**- *K. pneumoniae* positive control for *hlyA* and *hlyD* genes; **23**- 60; **24**- 64; **25**- 66; **26**- 72; **27**- 76; **28**- 78; **29**- 79; **30**- 86. Positive strains for *hlyA* and *hlyD* genes – 26; positive control.

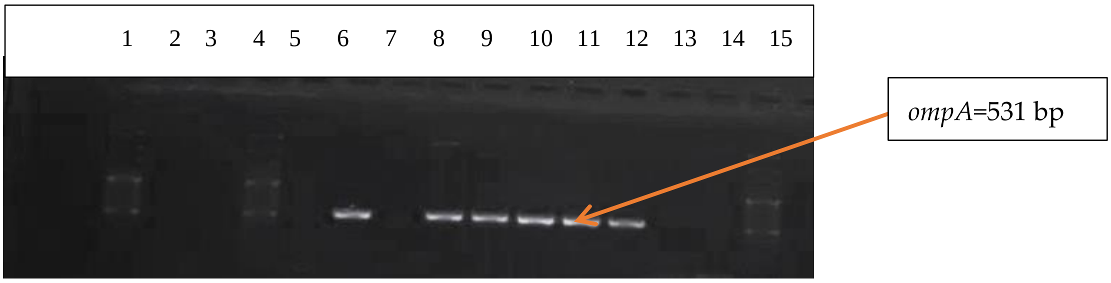

Figure S15. Electrophoresis gel for *ompA* (531 bp) gene detection in *A. baumannii* strains. Lines: **1**- Molecular Size Marker (ThermoScientific, 1500 bp); **2-** strain 35; **3-** 23; **4-** Molecular Size Marker (ThermoScientific, 1500 bp) ; **5-** Acc; **6-** *A. baumannii* positive control for *ompA* gene; **7-** 17; **8**- 21; **9**- 24; **10**- 27; **11-** 28; **12**- 49; **13**- 35; **14**- negativ control; **15**- Molecular Size Marker (ThermoScientific, 1500 bp). Positive strains for *OmpA* gene– positive control; 21; 24; 27; 28; 49.

Table S1. The resistance pattern of the isolated strains.

Microorganism	Source of isolation	Resistance to FQ, Carbapenems, Third generation Cephalosporins (% from total strains)	Resistance to TZP (% from total strains)	Resistance to AG (% from total strains)	Resistance to CZA (% from total strains)	Resistance to SXT (% from total strains)
K. pneumoniae	Urine	100	100	13.7 (AK)/ 21.6 (G)	9.8	41.2
	Blood culture	100	100	5.9 (AK)/ 11.8 (G)	5.9	11.8
	Other*	100	100	10 (AK)/ 11.9 (G)	2	21.6
Acinetobacter spp.	Urine	100	100	100		9.1
	Blood culture	100	100	100	NA	18.2
	Other*	100	100	100		27.3
P. aeruginosa	Urine	100	20	100	0	
	Blood culture	100	10	100	0	NA
	Other*	100	0	100	0	

*Biological fluids, wounds, endotracheal secretions, stool cultures, rectal swabs
Abbreviations- FQ= fluoroquinolones, TZP=Piperacillin/tazobactam, AG= Aminoglycosides, SXT= Trimethoprim/sulfamethoxazole, AK=Amikacin, G=Gentamycin, NA=not applicable (no strain was tested for this antibiotic)

Table S2. Virulence and β-lactamase encoding genes.

Microorganism	Virulence and β-lactamen coding genes	Urine N (%)*	Blood culture N (%)*	Bile Culture N (%)*	Wound N (%)*	Bronchial aspirate N (%)*	Rectal swab N (%)*	Drainage tube N (%)*	Ascitic fluid N (%)*	Stool culture N (%)*	p-value**
K. pneumoniae	*fimH*	4(15.4)	0	0	0	0	0	0	0	0	0.9
	fimA	13(50)	6(60)	0	0	2(50)	2(100)	0	0	0	0.275
	hlyD	2(7.7)	1(10)	0	0	0	0	1(50)	0	0	0.731
	hlyA	2(7.7)	1(10)	0	0	0	0	1(50)	0	0	0.731
	ecpRAB	23(88.5)	6(60)	1(100)	1(100)	4(100)	2(100)	1(50)	1(100)	2(66.7)	0.241
	mrkA	21(80.8)	8(80)	1(100)	1(100)	4(100)	2(100)	2(100)	1(100)	3(100)	0.947
	cnf1	5(19.2)	0	0	0	0	0	0	0	0	0.805
	bla_{KPC}	11(42.3)	1(10)	0	1(100)	2(50)	0	1(50)	1(100)	0	0.234
	bla_{TEM}	8(30.8)	5(50)	0	0	2(50)	2(100)	1(50)	0	1(33)	0.601
	bla_{NDM}	5(19.2)	3(30)	0	1(100)	0	0	0	0	0	0.460
	bla_{OXA}	5(19.2)	4(40)	0	0	1(25)	2(100)	1(50)	1(100)	1(33.3)	0.203
	bla_{SHV}	13(50)	3(30)	1(100)	1(100)	3(75)	0	1(50)	0	0	0.261
P. aeruginosa	*plcH*	3(75)	4 (100)	0	1(50)	0	0	0	0	0	0.335
	plcN	3(75)	4(100)	0	1(50)	0	0	0	0	0	0.335
	exoU	2(50)	3(75)	0	0	0	0	0	0	0	0.223
	exoS	1(25)	2(50)	0	1(50)	0	0	0	0	0	0.732
	exoT	3(75)	3(75)	0	1(50)	0	0	0	0	0	0.788
	algD	3(75)	4(100)	0	1(5)	0	0	0	0	0	0.335
	bla_{IMP}	1 (25)	0	0	0	0	0	0	0	0	0.435
	bla_{VIM}	2 (5)	0	0	0	0	0	0	0	0	0.153
Acinetobacter spp.	bla_{VIM}	0	1 (33)	0	0	0	0	0	0	0	0.817

bla$_{OXA-51}$	3(100)	3(100)	0	1(100)	1(100)	1(100)	1(100)	0	0	0.088
bla$_{OXA-23}$	0	3(100)	0	1(100)	1(100)	1(100)	1(100)	0	0	0.088
bla$_{OXA-24}$	1(33.3)	0	0	0	0	0	0	0	0	0.817
ompA	3(100)	3(100)	0	1(100)	1(100)	1(100)	1(100)	0	0	0.088

* N= number of isolates with a virulence gene or resistance gene. Percentages were calculated considering the total number of samples of a specific strain encountered in each isolation source. ** P-values reflected the significance between the differences in distribution of a virulence gene or resistance gene over the source types. Every p-value was obtained using a chi-test and only those values < 0.05 were considered significant. Abbreviation- NA=not applicable.
